# Prolonged Protective Immunity Induced by Mild SARS-CoV-2 Infection of K18-hACE2 Mice

**DOI:** 10.3390/vaccines10040613

**Published:** 2022-04-14

**Authors:** Liat Bar-On, Moshe Aftalion, Efi Makdasi, David Gur, Ron Alcalay, Hila Cohen, Adi Beth-Din, Ronit Rosenfeld, Hagit Achdout, Erez Bar-Haim, Reut Falach, Theodor Chitlaru, Ofer Cohen

**Affiliations:** 1 Biochemistry and Molecular Genetics Department, Israel Institute for Biological Research, Ness-Ziona 74100, Israel; moshea@iibr.gov.il (M.A.); gurd@iibr.gov.il (D.G.); rona@iibr.gov.il (R.A.); hilac@iibr.gov.il (H.C.); adib@iibr.gov.il (A.B.-D.); ronitr@iibr.gov.il (R.R.); erezb@iibr.gov.il (E.B.-H.); reutf@iibr.gov.il (R.F.); theodorc@iibr.gov.il (T.C.); 2 Infectious Diseases Department, Israel Institute for Biological Research, Ness-Ziona 74100, Israel; efim@iibr.gov.il (E.M.); hagita@iibr.gov.il (H.A.)

**Keywords:** SARS-CoV-2, COVID-19, K18-hACE2 mice, mild infection

## Abstract

Longevity of the immune response following viral exposure is an essential aspect of SARS-CoV-2 infection. Mild SARS-CoV-2 infection of K18-hACE2 mice was implemented for evaluating the mounting and longevity of a specific memory immune response. We show that the infection of K18-hACE2 mice induced robust humoral and cellular immunity (systemic and local), which persisted for at least six months. Virus-specific T cells and neutralizing antibody titers decreased over time, yet their levels were sufficient to provide sterile immunity against lethal rechallenge six months post-primary infection. The study substantiates the role of naturally induced immunity against SARS-CoV-2 infection for preventing recurring morbidity.

## 1. Introduction

The worldwide COVID-19 pandemic, caused by severe acute respiratory syndrome virus type two (SARS-CoV-2), has prompted numerous investigations addressing viral pathogenicity and host immune response. The longevity of the memory immune response following SARS-CoV-2 primary infection is a key parameter to understanding the protective immunity against reinfection with SARS-CoV-2 and establish the necessity of prophylactics against recurrent COVID-19. Clinical studies from convalescents have demonstrated a decrease in antibody titers and antigen-specific T cells over time [1,2,3,4]. Therefore, the possibility of reinfection is of significant public health concern. Furthermore, the effectiveness of the memory immune response following primary exposure in preventing reinfection remains elusive. The gaps in understanding these issues stem, in part, from the paucity of data regarding the progression of the immune response—in particular, in the lungs—from the onset of infection until the manifestation of immunological memory. The COVID-19 pandemic presents a broad spectrum of severities, ranging from asymptomatic presentation to severe pneumonia. Many studies have focused on immune responses induced by severe infections, but less is known about those induced by mild and asymptomatic infections, which account for a significant proportion of positive individuals [5,6].

SARS-CoV-2 infection of K18-hACE2 transgenic mice recapitulates COVID-19 pathogenesis [7]. In spite of the reported severe neurological condition of the transgenic mice [8], this model has been extensively used in studies addressing lethal infections, lung pathology, and the evaluation of antiviral prophylactics and countermeasures [9,10,11]. Yet, little is known about the duration and effectiveness of memory immunity following the primary infection of K18-hACE2 mice. In a few studies, immune responses were documented shortly after the resolution of infection, and therefore, they are not predictive of long-term memory and effectiveness in precluding reinfections [12,13].

In the current study, a mild SARS-CoV-2 primary infection model of K18-hACE2 mice was implemented for assessing the establishment of a specific memory response and its efficacy in protecting against a subsequent lethal challenge. Primary asymptomatic infection induced a robust adaptive immune response, including the generation of neutralizing antibodies and virus-specific T cells (systemic and local). The response persisted for at least 6 months, as, to the best of our knowledge, was documented for the first time. We demonstrated that, although the magnitude of the memory immune response declined over time, it was sufficient to provide sterile immunity against lethal rechallenge as late as 6 months post-primary infection.

## 2. Materials and Methods

### 2.1. Cells and Viruses

Vero E6 cells (ATCC^®^ CRL-1586TM) were maintained in Dulbecco’s modified Eagle’s medium (DMEM) supplemented with 10% fetal bovine serum (FBS), MEM nonessential amino acids, 2 nM L-glutamine, 100 units/mL penicillin, 0.1 mg/mL streptomycin and 12.5 units/mL nystatin (Biological Industries, Israel). The cells were cultured at 37 °C in a 5% CO_2_ and 95% air atmosphere.

SARS-CoV-2 (GISAID accession EPI_ISL_406862) was kindly provided by Bundeswehr Institute of Microbiology, Munich, Germany. Stocks were prepared and tittered by infection of VeroE6 cells as described [14]. The virus was aliquot and stored at −80 °C until use.

### 2.2. Animal Experiments

All animal experiments were conducted in a biosafety level 3 (BSL3) facility. The animal handling was performed in accordance with the regulations outlined in the U.S. Department of Agriculture (USDA) Animal Welfare Act and the conditions specified in the Guide for Care and Use of Laboratory Animals (National Institutes of Health, Bethesda, MD, USA. 2011). The animal studies were approved by the ethical committee for animal experiments of the Israel Institute for Biological Research (IIBR) (protocol number M-13-21). Female K18-hACE2 transgenic mice (B6.Cg-Tg(K18-ACE2)2Prlmn/J; #034860) (Jackson Laboratory, Bar Harbor, ME, USA) (6–8 weeks old) were maintained at 20–22 °C and a relative humidity of 50 ± 10% under a 12-h light/dark cycle. The animals were fed commercial rodent chow (Koffolk Inc., Ramat Hovav, Israel) and provided tap water ad libitum. Prior to infection, the mice were kept in groups of 10. The mice were randomly assigned to the experimental groups.

For SARS-CoV-2 infection, the virus was diluted in phosphate-buffered saline (PBS) supplemented with 2% FBS (Biological Industries, Beit Haemek, Israel). Anesthetized animals (ketamine 75 mg/kg and xylazine 7.5 mg/kg in PBS) were infected by i.n. instillation of 30 µL (80 PFU/mouse). In all experimental groups, 30% of the animals succumbed following the initial infection (80 PFU/mouse). The remaining 70% animals did not disclose any detectable sign of disease and served for the study. Body weight and clinical signs of disease were monitored daily throughout the follow-up period post-infection.

Lethal challenge of the animals at the various times post-primary infection was carried out by administration of 3 × 10^4^ PFU/mouse.

### 2.3. ELISA

Direct ELISA was performed for the detection of SARS-CoV-2-specific IgG antibodies in mouse sera as previously described [14]. Recombinant spike-RBD protein (2 μg/mL; [11]) was used for plate coating. Dil_50_ was defined as serum dilution at which 50% maximum binding was achieved.

### 2.4. ELISpot Assays

IFN-γ ELISpot to spleen or lung cells was done as described [14]. The spleens were dissociated in GentleMACS C-tubes (Miltenyi Biotec, Bergisch Gladbach, Germany), filtered and separated on Ficoll-Paque (GE). Lungs were cut and digested with collagenase D (Roche, Mannheim, Germany) (4 mg/mL, 1 h, 37 °C). Then, 4 × 10^5^ cells from each sample were plated into 96-well ELISpot plates in duplicate in the presence of commercial SARS-CoV-2 S1 peptide pool (2µg peptide/mL; milteny) and incubated at 37 °C for 24 h. The frequency of IFNγ-secreting cells was determined using a Mouse IFN-γ single-Color ELISpot kit (Cellular Technology Limited, Biotec, Bonn, Germany) according to the manufacturer’s instructions. The frequency of cytokine-secreting cells was quantified with an ImmunoSpot S6 Ultimate reader and analyzed with ImmunoSpot software (Cellular Technology Limited, Bonn, Germany). Antigen-free cells supplemented with medium were used as a negative control.

### 2.5. Determination of the Viral Load in Organs

The viral loads were determined at 2 and 4 days post-infection. The lungs and brains were harvested and stored at −80 °C until further processing. The organs were processed for titration in 1.5 mL of ice-cold PBS. Tissues were homogenized (ULTRATURAX^®^ IKA R104) for 30 s in 1.5 mL of ice-cold PBS, followed by centrifugation (270× *g*, 10 min, 4 °C) and collection of the supernatants for analysis of the viral load. The SARS-CoV-2 viral load was determined using a PFU assay [14]. Serial dilutions of extracted organ homogenates from mice infected with SARS-CoV-2 were prepared in the infection medium (MEM containing 2% FBS) and used to infect Vero E6 monolayers in duplicate (200 µL/well). The plates were incubated for 1 h at 37 °C to allow viral adsorption. Then, 2 mL overlay (MEM containing 2% FBS and 0.4% tragacanth; Merck, Rehovot, Israel) was added to each well, and the plates were incubated at 37 °C in a 5% CO_2_ atmosphere for 48 h. The medium was then aspirated, and the cells were fixed and stained with 1 mL/well crystal violet solution (Biological Industries, Beit Haemak, Israel). The number of plaques per well was determined, and the SARS-CoV-2 PFU titer was calculated.

### 2.6. Plaque Reduction Neutralization Test

The SARS-CoV-2-neutralizing antibody levels were determined using a plaque reduction neutralization test (PRNT) with SARS-CoV-2 as previously described [11]. Briefly, all sera were heat-inactivated at 60 °C for 30 min, then serially diluted twofold in 400 µL of infection medium, mixed with 400 µL of 300 PFU/mL SARS-CoV-2 and incubated at 37 °C in an atmosphere of 5% CO_2_ for 1 h. Then, 200 μL of each serum/virus mixture was added in duplicate to Vero E6 cell monolayers, and the cells were incubated for 1 h at 37 °C. A virus mixture without serum was used as a control. Two milliliters of overlay was added to each well, and the plates were incubated at 37 °C in a 5% CO_2_ atmosphere for 48 h. The medium was then aspirated, and the cells were fixed and stained with 1 mL/well crystal violet solution. The number of plaques per well was determined, and the serum dilution that neutralized 50% of the virions (NT_50_) was calculated using Prism software (GraphPad Software Inc., San Diego, CA, USA).

### 2.7. Quantitative Real-Time RT-PCR

RNA was extracted with a viral RNA mini kit (Qiagen, Hilden, Germany) according to the manufacturer’s instructions. Quantitative real-time RT-PCR was conducted with a SensiFAST™ Probe Lo-ROX One-Step Kit (Bioline, Memphis, TN, USA) and analyzed with a 7500 Real-Time PCR System (Applied Biosystems, Bedfprd, MA, USA) using E-specific primers [15].

### 2.8. Statistical Analysis

Data were analyzed using GraphPad Prism Software version 9.2.0 (GraphPad Software, San Diego, CA, USA). Statistical analysis was performed using one-way ANOVA with Tukey’s multiple comparison test, assuming normal distribution. The line represents the means, and the error bar represents the SDs. *p*-values indicate significant differences (* *p* < 0.05; ** *p* < 0.005; *** *p* < 0.0005; **** *p* < 0.0001).

## 3. Results

### 3.1. Persistence of the Immune Response Post Asymptomatic SARS-CoV-2 Infection

**Figure 1:**
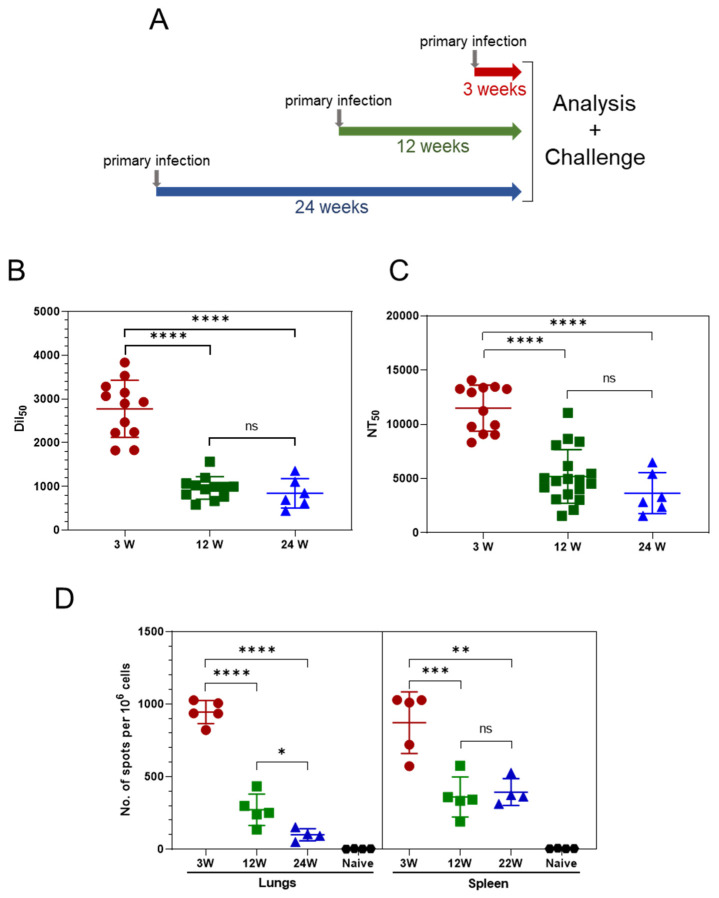
Humoral and cellular-specific immune response after mild SARS-CoV-2 infection. K18-hACE2 mice were infected with SARS-CoV-2 (80 PFU/mouse, i.n.) 24 weeks (blue), 12 weeks (green) or 3 weeks (red) prior to their simultaneous analysis and challenge. Sera, spleen and lung samples were collected at the indicated time points for the evaluation of SARS-CoV-2-specific humoral (**B**,**C**) and cellular (**D**) responses, as describe in Materials and Methods. (**A**) Schematic representation of experimental design. (**B**) SARS-CoV-2 RBD-specific IgG antibodies in the serum of the 3 groups prior to the challenge, determined by ELISA. (**C**) NT_50_ titers determined by the Plaque Reduction Neutralization test (PRNT). (**D**) SARS-CoV-2-specific cellular response determined by ELISpot in the lungs (left panel) and spleen (right panel). Each symbol represents one mouse. *p*-values indicate significant differences (* *p* < 0.05; ** *p* < 0.005; *** *p* < 0.0005; **** *p* < 0.0001).

K18-hACE2 mice were intranasally inoculated with a low dose of 80 PFU SARS-CoV-2, 24 weeks, 12 weeks or 3 weeks prior to their simultaneous analysis and rechallenge (see scheme in Figure 1A). Following the initial infection, approximately 30% of the animals in all the experimental groups succumbed. Most importantly, the other 70% of the animals did not exhibit any visible disease symptoms such as weight loss, rough hair, decreased activity, or loss of social behavior (data not shown). These asymptomatic animals served for the study.

Serum samples were collected from the three experimental groups, and the humoral response was quantified by anti-IgG RBD-specific ELISA. High levels of IgG were detected three weeks following SARS-CoV-2 inoculation (Figure 1B). However, a significant decrease in antibody levels was observed at 12 weeks, yet remained stable, as determined 24 weeks after infection. A plaque reduction neutralization test (PRNT) using the SARS-CoV-2 virus (Figure 1C) was implemented to determine the titers of neutralizing antibodies (NAb). As demonstrated, high NAb titers were observed 3 weeks post-infection and declined sharply in the following weeks. As in the case of the IgG titers, the level of NAb remained relatively stable between 12 and 24 weeks post-infection. Thus, asymptomatic SARS-CoV-2 exposure elicited robust and long-lived humoral responses in K18-hACE2 mice, exhibiting an initial decrease followed by a steady level.

Next, we examined whether this mild infection with SARS-CoV-2 elicited cellular immunity and evaluated their persistence. The SARS-CoV-2-specific T-cell response was assessed by employing an IFNγ ELISpot assay. High numbers of specific T cells in the spleens (500–1500 spots per 10^6^ splenocytes; Figure 1D) were detected 3 weeks post-infection. Twelve weeks post-infection, the number significantly declined (100–500 spots per 10^6^ splenocytes). The frequency of splenic SARS-specific T cells remained steady 24 weeks after infection (12 weeks later), indicative of the persistence of systemic memory T cells. The local T-cell response in the lungs was characterized by high numbers of SARS-specific T cells 3 weeks post-infection (~1000 specific cells per 10^6^ lung cells), which declined significantly 12 weeks post-infection. In contrast to the systemic T-cells pool, which remained stable at 24 weeks, the number of T cells measured in the lungs 24 weeks post-infection continued to decline (Figure 1D), yet their level was still significant. Taken together, these results indicate that asymptomatic SARS-CoV-2 exposure elicited a robust SARS-CoV-2-specific T-cell response both systemically and in the lungs.

### 3.2. Primary SARS-CoV-2 Infection Protects K18-hACE2 Mice against Reinfection

We investigated the ability of the memory immune response to protect against a lethal challenge with SARS-CoV-2 at the different time points following the first infection.

**Figure 2:**
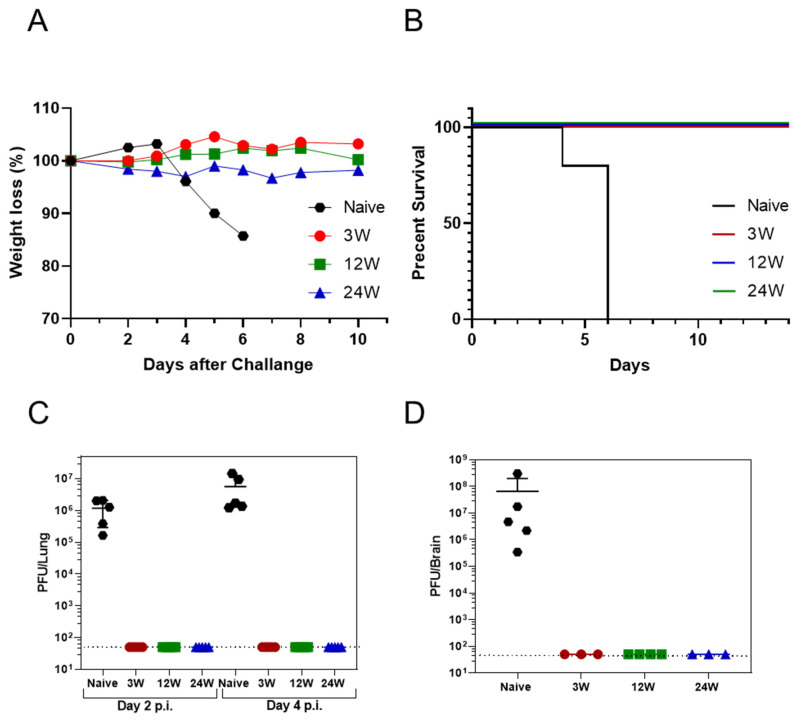
Protective efficacy against SARS-CoV-2 reinfection. K18-hACE2 mice from the 3 groups, as indicated (see Figure 1A, *n* = 20 for each group), were reinfected i.n. with 3 × 10^4^ PFU SARS-CoV-2. Control group consisted of naïve animals (*n* = 15). Animals were monitored daily for body weight (**A**) and survival (**B**). Five mice from each group were sacrificed at day 2 and 4 post-challenge. SARS-CoV-2 loads in the lungs (**C**) and brain (**D**) were determined by a PFU assay. Data points represent the virus load of each animal; the horizontal bar indicates the mean of each group. The dashed line indicates the lower limit of detection. Statistical analysis was performed with the log-rank (Mantel–Cox) test for survival data.

The three groups of K18-hACE2 mice (3/12/24 weeks) were simultaneously infected intranasally with high doses of the virus (3 × 10^4^ PFU; 375 times the initial dose). Animal weight and survival were monitored for 10 days post-infection (Figure 2A,B, respectively). As demonstrated, naïve control group animals exhibited significant weight loss beginning 4 days post-infection (Figure 2A) and succumbed 6 days post-exposure (Figure 2B). Most notably, all of the previously exposed mice (10/10 for each group), regardless of the time from the initial SARS-CoV-2 exposure, were fully protected against the recurring challenge and did not exhibit any weight loss (Figure 2A). Furthermore, the viral load in the lungs of the infected mice was examined by a viral plaque assay at days 2 and 4 following the second exposure. As early as 2 days post-exposure, no infectious virions were detected (by the plaque assay) in the lungs of previously exposed mice (Figure 2C), whereas high levels of infective virus were present in the naïve control mice (10^5^–2 × 10^6^ PFU/organ). High levels of the virus were also detected in the naïve mice lungs (10^6^–5 × 10^7^ PFU/organ) and brains (3 × 10^5^–3 × 10^8^ CFU/organ) 4 days after the challenge, while no virus was detected in previously exposed mice (Figure 2D). The viral burden monitored by the plaque assay 2 and 4 days after the challenge was further confirmed by a specific qRT-PCR assay that did not detect viral genomic RNA in the lungs and brains of the previously infected mice (data not shown). These data strongly support the conclusion that SARS-CoV-2 mild infection of K18-hACE2 mice resulted in elicitation of a memory immune response that prevented with high efficacy a high-dose re-infection as late as 6 months following the primary exposure.

## 4. Discussion

Determining the durability of the immune response of convalescent COVID-19 individual infections is of outmost importance for fully understanding the host response to SARS-CoV-2 infection and represents essential information for the public health management of the pandemic [16,17,18,19].

In the current study, we evaluated the longevity and the potency of the immune responses elicited by primary infection with SARS-CoV-2 using the K18-hACE2 mouse model. We demonstrated that mild and asymptomatic infection results in robust immunity lasting at least six months. This observation strengthens the notion that SARS-CoV-2 is highly immunogenic and suggests that the disease itself and the accompanying severe inflammatory conditions are not essential for long-lasting immunity to SARS-CoV-2. The present report documents that the immune response triggered by exposure to the virus consists of both systemic and local memory T cells, as well as circulatory neutralizing antibodies. Based on our findings, K18-hACE2 mice (which were mainly employed for addressing fulminant manifestations of the disease) are also appropriate for studying the immune memory following mild SARS-CoV-2 infection both at the humoral and cellular levels. Of note, the low dose administered to the mice is not associated with the complication of the model related to the presence of the virus in the central nervous system (presence of the virus in the brains of the infected mice was rule out by direct analysis, data not shown).

We demonstrated that, after the initial 3 weeks post-infection, the number of specific T cells and antibody levels decreased, as seen 12 weeks post-infection, consistent with an early transient effector phase of the immune response. The contraction phase is followed by a period of at least 12 weeks during which neither the NAb titers nor the SARS-CoV-2-specific systemic T cells decreased significantly, indicating that, during this period, a stable memory response is maintained. In contrast to the stable number of systemic SARS-CoV-2-specific T cells detected in the spleen 24 weeks post-infection, the population of local SARS-CoV-2-specific T cells continued to decline in the lungs. This observation is in line with the notion that lung resident memory T cells wanes within few weeks following other viral or bacterial infections [20].

Understanding the complexity of immune memory against SARS-CoV-2 is important to gain insight into the likelihood of durable immunity against reinfection with SARS-CoV-2 and secondary COVID-19 disease. Numerous studies are being conducted using animal models to determine the relative contribution of neutralizing Abs and memory T responses in protecting against SARS-CoV-2 infection [12,21,22,23,24,25,26]. However, possible mechanisms of immunological protection can vary based on the kinetics of the immune memory responses, the infection and the selected model [3].

One of the most important observations reported in this communication is that six months post-non-symptomatic infection, the previously exposed mice were fully protected against a recurring infection involving exposure to a highly lethal dose of SARS-CoV-2. Notably, six months following mild infection, a complete virus neutralization was observed, as early as in the second day post reinfection.

While we cannot rule out the possibility that the early viral clearance involves rapid induction and deployment of local lung resident memory T cells, full and sterile immunity, as determined in the present study, is usually mediated by neutralizing antibodies [27]. Indeed, 6 months post-mild infection, stable amounts of neutralizing antibodies persist in circulation, and their titers may be sufficient to provide sustained sterile immunity. However, the efficiency of the response against recurring reinfection with newly emerging variants, exhibiting significant antigenic variation, will require further studies.

## 5. Conclusions

In this study we demonstrated that (1) mild and asymptomatic SARS-CoV-2 infection in K18-hACE2 mice conferred robust humoral and cellular immune responses that persisted for at least six months; (2) regardless of the time from the initial SARS-CoV-2 exposure, all previously exposed mice were fully protected against recurring infection. Furthermore, the mice appeared to have developed sterile immunity, as evidenced by the fact that no infectious viruses were detected following a lethal challenge.

The study highlights the strength of natural infections in generating long-lasting immunity in the K18-hACE2 transgenic mouse model.

## Data Availability

All raw data associated with the study are available upon request from corresponding authors.
